# Mounted Smartphones as Measurement and Control Platforms for Motor-Based Laboratory Test-Beds †

**DOI:** 10.3390/s16081331

**Published:** 2016-08-20

**Authors:** Jared A. Frank, Anthony Brill, Vikram Kapila

**Affiliations:** Department of Mechanical and Aerospace Engineering, NYU Tandon School of Engineering, Mechatronics and Control Laboratory, Brooklyn, NY 11201, USA; jared.alan@nyu.edu (J.A.F.); brill.anthony@nyu.edu (A.B.)

**Keywords:** control, laboratory, mechatronics, sensing, smartphone

## Abstract

Laboratory education in science and engineering often entails the use of test-beds equipped with costly peripherals for sensing, acquisition, storage, processing, and control of physical behavior. However, costly peripherals are no longer necessary to obtain precise measurements and achieve stable feedback control of test-beds. With smartphones performing diverse sensing and processing tasks, this study examines the feasibility of mounting smartphones directly to test-beds to exploit their embedded hardware and software in the measurement and control of the test-beds. This approach is a first step towards replacing laboratory-grade peripherals with more compact and affordable smartphone-based platforms, whose interactive user interfaces can engender wider participation and engagement from learners. Demonstrative cases are presented in which the sensing, computation, control, and user interaction with three motor-based test-beds are handled by a mounted smartphone. Results of experiments and simulations are used to validate the feasibility of mounted smartphones as measurement and feedback control platforms for motor-based laboratory test-beds, report the measurement precision and closed-loop performance achieved with such platforms, and address challenges in the development of platforms to maintain system stability.

## 1. Introduction

The pedagogical rationale for hands-on laboratory explorations in science and engineering education is well established and includes improvement in students’ conceptual understanding as well as opportunities to hone design, professional, and social skills [1]. However, instructional laboratories are increasingly burdened by the costs of purchasing and maintaining equipment. Test-bed peripherals, including hardware and software used to collect and process experimental measurements, can often contribute to significant costs. Efforts to provide valuable educational experiences that avoid such large costs have included the deployment of virtual laboratories that operate on simulations of physical processes, thus obviating the need for sensing, actuation, and data acquisition [2]. An alternative approach has allowed students to remotely perform experiments and collect data from test-beds using a web browser, reducing the number of test-beds required at the laboratory site and allowing students “anytime, anywhere” access to laboratory-grade equipment [3]. Despite the advantages of virtual and remote labs, studies suggest that the loss of physical presence with actual equipment frequently causes learners to: have difficulty in achieving learning outcomes, experience lack of motivation, and express preference for hands-on labs [4]. To provide cost-effective, readily-accessible solutions that allow measurement, control, and enhanced interaction with real equipment, developers of platforms for science and engineering courses, as well as educators of such courses, can leverage the smartphones that learners already bring with them to the laboratory.

The pervasive adoption of smartphones has launched a new era of personal computing in which the ability to sense, store, process, and communicate information is available in the palm of one’s hand. The sensing, storage, computation, and communication (SSCC) capabilities of smartphones have enabled them to serve as lightweight, portable, and inexpensive platforms for a variety of measurement applications [5]. For example, the inertial sensors of smartphones have been used for positioning applications in indoor environments where GPS signal is weak [6] and to encourage independent navigation of the visually impaired [7]. Mobile applications have analyzed inertial data to recognize user activity, e.g., walking, jogging, and ascending or descending stairs [8]. In the medical field, applications include processing and integrating data from smartphone inertial sensors to measure the Cobb angle in both kyphoses [9] and scoliosis [10], analyze gait characteristics [11], characterize Parkinson’s disease tremor [12], and notify emergency services if a user has fallen [13]. A smartphone implementation may also yield cost reductions *vis-a-vis* conventional hardware. For instance, a system that processes accelerometer data from a smartphone to detect potholes has been proposed as a substitute for high-cost equipment [14]. A study that used a smartphone to detect traffic collisions and notify emergency services [15] noted that similar in-vehicle systems are infeasible to retrofit into older vehicles and too expensive to include in newer vehicles.

Several innovative mobile applications have leveraged smartphone cameras for vision-based measurement, e.g., to recognize fingerprints [16], to enable farmers to remotely classify fish species [17], and to assist the visually impaired in both indoor and outdoor navigation [18]. Moreover, a system has been developed that fuses information from several different smartphone sensors, including both front- and rear-facing cameras, to monitor driving conditions [19]. Computer vision and machine learning algorithms are performed on the phone to detect the head pose and eye states of the driver to infer his/her attentiveness and drowsiness during driving, as well as to detect unsafe conditions such as tailgating, lane weaving, or drifting. It has been noted that similar built-in features are only found in the most expensive of cars [19].

The use of smartphone-collected sensor data in the closed-loop networked control of physical systems remains largely unexplored. A notable exception is the construction of an artificial pancreas system using a smartphone to process wireless data from a continuous glucose monitoring system worn by patients with type 1 diabetes [20], improving the mobility of users compared to previous prototypes, which were tethered to a PC. Additional examples include smartphone-controlled robots and unmanned vehicles, in which the multi-core processors and real-time operating systems of smartphones have been leveraged to handle large computational loads [21]. Mounted smartphones have been used for obstacle avoidance of a marine vehicle [22] and for the stabilization and control of unmanned aerial vehicles [23]. These studies use the device camera to capture video and computer vision techniques such as template matching, feature tracking with random sampling consensus, and color segmentation to enable the autonomous guidance and navigation of the vehicle. In this role, smartphones use embedded sensors to detect obstacles in the environment, sense collisions, estimate the pose of the vehicle, and compute vehicle velocities.

Advancements in mobile technology provide a unique opportunity for the integration of smartphones into test-beds for science and engineering laboratories. Specifically, there exist test-beds that are amenable to housing a mounted smartphone that performs some of the required sensing, control, and user interface tasks. Such smartphone-mounted laboratory test-beds (SMLTBs) can enable learners to quickly and seamlessly perform experiments with increased portability and reduced cost. Moreover, having learners mount their personal devices onto equipment and interact with the test-bed through an interface on a mobile application can stimulate engagement in laboratory work, changing learners’ views of their devices from consumer products to technological tools.

Despite their promising potential, the development of SMLTBs presents several challenges related to the mounting of the smartphone, modeling and designing the controller for the SMLTB, and programming the back end (computing) and front end (user interface) of the mobile application. This study investigates the factors that can have a significant impact on the stability of the SMLTB’s physical dynamics and the responsiveness of the user interface, and describes guidelines and efficient algorithms to address them for three illustrative SMLTBs in which mounted smartphones provide sensing, control, and user interfaces for the system. These three SMLTBs are based on classic platforms that exhibit rotational and/or translational motion and have consistently been used in feedback control research and education, as well as in investigations of new technologies for feedback control. Specifically, the study explores the position control of a DC motor, a naturally stable system whose full state is measured directly from a smartphone’s inertial sensors, the use of an efficient vision-based technique to balance an inverted pendulum on a cart (IPC), a naturally unstable and underactuated system that imposes stricter demands on processing time and sampling rate than the motor, and the integration of both inertial and vision-based sensing to stabilize a ball and beam, an even more complex system that requires a multi-rate estimation and control approach. For each case, a discussion is provided on the smartphone mounting, sensing approach, system model, control design, and user interface. Simulations and experiments are conducted to validate the feasibility of mounted smartphones as measurement and feedback control platforms for engineering laboratories, to report the measurement precision and closed-loop performance achieved with such platforms, and to address major challenges and considerations encountered in the development of the platforms.

## 2. Motivation: Smartphones as Laboratory Tools

As society experiences changes in the economic and technological landscape, educational institutions are expected to adapt the format of their laboratory instruction. For institutions with limited resources, maintaining hands-on laboratories has posed a serious economic burden. Meanwhile, the potential of mobile hardware and software to deliver valuable educational experiences is beginning to be explored and is inspiring novel educational technology formats, e.g., mobile learning [24] and mobile-assisted seamless learning [25]. Development of novel mobile applications is beginning to transform smartphones into powerful tools for science education, allowing students to collect, analyze, and share data right from the devices in their pockets both in and out of classrooms [26]. Recent advances include educational applications that utilize the capabilities of mobile devices to provide learning experiences fueled by interactive augmented reality [27], simulated physical dynamics [28], real-world measurement and portable experimentation [29], and mobile access to remote laboratories [30]. Thus, the SSCC capabilities of mobile devices have been leveraged to different degrees to provide learners with the information, tools, data, visualizations, measurements, and remote access needed for effective learning.

A majority of people now own smartphones. Since students and educators already bring smartphones into the laboratory with them, large costs can be eliminated by replacing laboratory-grade sensing and PC-based DAC hardware, which can cost thousands of dollars per laboratory station, with these devices, which institutions will not need to purchase. For a diverse array of laboratory test-beds, a smartphone can be readily mounted and its SSCC capabilities integrated to measure and control the state of the test-bed while hosting a user interface for interaction with the test-bed. This is because, due to advances in MEMS technology and the availability of affordable and compact cameras, smartphones can provide measurements that are comparable to those from laboratory-grade sensors in some feedback control applications. Moreover, smartphones provide the capacity to develop graphically rich, intuitive user interfaces that may be distributed to students to enhance their interactions with test-beds. In spite of their promising potential, no study has yet investigated smartphone-mounted solutions for measurement and feedback control of laboratory test-beds.

## 3. Smartphone-Mounted Laboratory Approach

When attempting to visualize and develop a deeper understanding of abstract concepts, students benefit greatly by seeing and interacting with concrete, physical demonstrations [31]. In engineering fields such as automatic control, laboratory test-beds are developed that often undergo some form of motion [32]. Fortunately, several different technologies that can sense motion are housed in smartphones. Laboratory test-beds that display rotational or translational motion are thus amenable to being fitted with smartphones that serve as platforms for contactless sensing and wireless control of the test-beds. The suggested implementation has the advantages of reduced wiring, lower cost, and smaller form factor. Moreover, allowing learners to leverage their personal devices in the experimental process can provide them with more engaging and convenient laboratory experiences.

### 3.1. Mounting a Smartphone

In securing a smartphone to a test-bed so that its embedded sensors can be used to obtain accurate measurements of the system’s state, it is critical to examine where and how the smartphone is to be mounted. At the scale of the cases considered in this study, the added volume and inertia of the mounted smartphone is not negligible. In fact, the placement of the smartphone will affect several other stages of the development, including the modeling of the system and design of the controller. Moreover, the placement of the smartphone needs to be chosen with consideration for the sensing approach to be taken. It will be shown that if the placement and sensing strategies as well chosen, then subsequent phases of development can be simplified considerably.

### 3.2. Sensing Modalities

While deciding the placement of the smartphone on the test-bed, developers are confronted with the question of how the smartphone will be used to capture the state of the system. Mobile applications have been developed that utilize the embedded sensors of smartphones to monitor a wide variety of user activities and physical states [33]. Some of the most powerful and popular of these sensors are the inertial sensors and digital cameras. In this study, both of these sensing modalities are considered for measuring the motions exhibited by the test-beds.

#### 3.2.1. Inertial Measurement

Inertial measurement units (IMUs) have become a standard sensor on board smartphones, often being used in applications with gesture-based interactions. They consist of 3-axis gyroscopes, accelerometers, and magnetometers, whose raw data can be used directly or processed by sensor fusion algorithms to estimate the attitude of the device. Device attitude is represented with Euler angles and defined as the rotation between the device’s current reference frame and the initially established reference frame when the mobile application is initialized. By rigidly mounting the smartphone to a test-bed, the attitude readings of the smartphone indicate the attitude of the test-bed. Since the test-beds considered in this study exhibit planar motion, only orientation about one axis is of interest. Choosing the rate that the IMU is sampled at is an important design consideration since it has a significant impact on the system model, the controller design, and the performance of the closed-loop system. The mobile applications developed in this study support IMU sampling rates of up to 100 Hz to detect high-frequency motions such as impacts and quick shakes. However, developers must keep in mind that although a faster sampling rate yields a more stable system response, it significantly increases battery consumption.

Because the smartphone’s attitude estimates are relative measurements that tend to drift with time, calibration of the readings is an important consideration. Calibration of attitude estimates can be performed using either the smartphone’s magnetometer, which is a source of absolute measurement, or an inexpensive external sensor such as a potentiometer or encoder. For the purposes of evaluating characteristics of measurements collected on the smartphone (e.g., accuracy, noise, drift), in this paper, experimental data from the smartphone-based measurement is compared with corresponding data from potentiometers and encoders embedded on the test-beds. The experiments are initiated at the orientation corresponding to the external sensor’s zero measurement. Thus, the angular position readings from the smartphone and the external sensors can be compared.

#### 3.2.2. Vision-Based Measurement

Vision-based sensing offers a data-rich, affordable, contactless sensing mechanism for many measurement and control applications. Practically all modern smartphones now have integrated digital cameras that can capture high resolution video at frame rates as high as 60 fps (frames per second). Thus, smartphones are better equipped than ever to run fundamental computer vision algorithms that capture, in real time, a physical system’s state, which can be processed by the feedback control algorithms to apply actuation signals on the system. To extract system state, two main vision approaches can be used. The first approach utilizes algorithms that work with the pixel coordinates of features in the image. In the second approach, a calibrated camera is used to estimate the pose between the camera’s coordinate system and a real-world coordinate system so that algorithms can work with real-world coordinates of features in the scene. In this study, both image-based and pose-based approaches are explored in the stabilization of test-beds. Due to inherent nonlinearities, underactuation, large bandwidth, and open-loop instability of many automatic control test-beds, vision-based control of such systems poses challenging demands on processing time, frame rate, and image quality. Thus, to implement SMLTBs successfully, it is critical to investigate the effects of these parameters on the stability and performance of the systems.

### 3.3. System Modeling and Control Design

Because mounting a smartphone to the test-bed adds a significant inertia, which must be driven by the motor, the test-bed dynamics needs to be remodeled to take into account the presence of the smartphone. If the smartphone is placed such that it is aligned with the motor shaft, the new model will be relatively simple. However, as will be shown, the farther from the rotational axis the smartphone is placed, the larger will be the effects of nonlinear terms in the dynamic model. This can necessitate a change in the control algorithm needed to stabilize the SMLTB. Furthermore, a smartphone located far from the rotational axis contributes a large load on the motor, destabilizing the system and making control more difficult.

### 3.4. Communication

Once the application running on the smartphone has collected and processed all necessary measurements, it must communicate this data as feedback for driving the test-bed. This communication is chosen to be wireless to avoid cable entanglement that can result from the rotating smartphone. Communication of data between the smartphone and an external processor that drives the test-bed is performed over Wi-Fi using a client-server architecture. During experimentation, to validate the use of SMLTB, the client is executed on the smartphone and the server is executed on a PC and accessed via a wireless local area network maintained by a router. Data is sent and received using the TCP/IP protocol. To allow data to be sent and received in real time, the Nagle algorithm, which is built into the TCP/IP protocol to improve the efficiency of communication [34], is disabled on both sides of the communication because it introduces latency.

### 3.5. User Interface Design

Mounting smartphones to laboratory test-beds not only allows for its on-board hardware to monitor and control the system, but also allows its software to provide an interactive user interface mounted directly to the experiment. In designing the front end of the mobile application, developers have the opportunity to present learners with educational content; instructions for the experimental process; and natural controls for adjusting system parameters, commanding the test-bed to a desired state, and saving collected data for post-processing. As the interface is the focus of the user’s attention and the means by which s/he interacts with the test-bed and conducts experiments, its design is critical to the quality of the user experience with the SMLTB.

## 4. Example Test-Beds

All the test-beds discussed in this study are based on motorized platforms whose rotational or translational motion can be controlled using measurements from a mounted smartphone. These smartphone-mounted systems are investigated with respect to the smartphone’s ability to adequately measure and estimate the system state such that the control of the system in a wireless feedback loop exhibits acceptable performance characteristics. In this study, each test-bed is mounted with an iPhone. For the last few years more than 95% of the smartphone market has been shared almost equally between Android and iOS devices, which for the most part have employed similar peripheral hardware (including inertial sensors from STMicroelectronics, AKM Semiconductor, and Bosch Sensortec and high resolution cameras from manufacturers like Sony) and software support. Thus, feasibility conclusions of the study are expected to be valid for both classes of devices.

### 4.1. DC Motor Test-Bed

**Figure 1:**
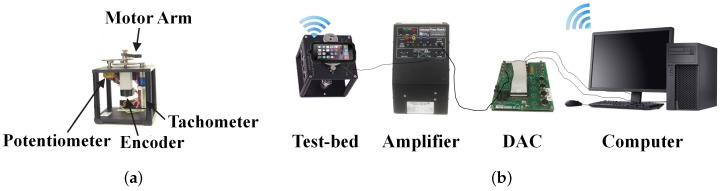
(**a**) DC motor test-bed and (**b**) experimental setup with a PC-based DAC.

The first test-bed is a DC motor housed in an enclosure with a gearbox and several sensors to measure its rotational motion, i.e., an incremental optical encoder and a multi-turn potentiometer to measure orientation and a tachometer to measure angular rate (see Figure 1a). A motor arm is attached to the output shaft of the gearbox and is driven by a power amplifier, which receives control signals from a personal computer (PC) via a PC-based data acquisition and control (DAC) board. An Apple iPhone 5, which contains a three-axis accelerometer and gyroscope from STMicroelectronics and a magnetometer from AKM Semiconductor, is used to capture the state of the motor arm so that control signals can be computed and sent to the PC over Wi-Fi, as shown in Figure 1b. Although the iPhone 5 was discontinued three years prior to the publication of this paper, it is used to investigate the performance that would be achieved for end users who typically own older generation devices.

#### 4.1.1. Smartphone Measurement

**Figure 2:**
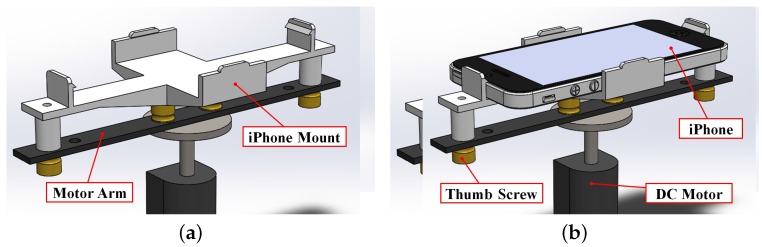
Design of a mount for the DC motor (**a**) without and (**b**) with a smartphone secured.

In this first case, the only sensing required is that of the motion of the motor arm about the axis of the output shaft of the gearbox. To minimize the additional inertial load on the motor, the smartphone is placed such that one of its centroidal axes is aligned with the rotational axis of the motor shaft. A mount is designed and 3D-printed (as shown in Figure 2a) using guidelines and dimensions provided by the manufacturer of the smartphone, with four tabs to rigidly hold the phone in place and prevent it from rotating with respect to the motor arm (see Figure 2b). To allow users to interact with the test-bed using the front end of the mobile application, the mount is designed such that the buttons and screen of the smartphone are not blocked. In this configuration, device orientations are measured using the smartphone’s inertial sensors about the direction normal to the device screen and provide the orientation of the motor arm.

#### 4.1.2. System Modeling and Control

**Figure 3:**
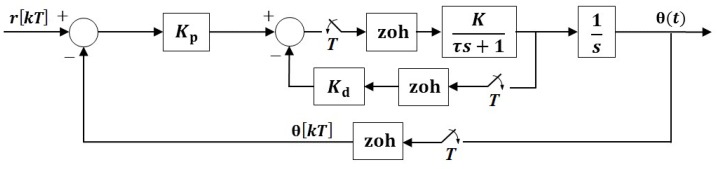
Sampled-data block diagram of the closed-loop motor control system.

To control the orientation of the motor arm using a mounted smartphone, the mobile app on the smartphone computes the control action u(t) at time t=kT, where *T* is the sampling rate and k=0,1,2,…, after the angle θ[kT] and angular rate ω[kT] have been sampled. This control computation is done according to the proportional and derivative (PD) control law $u\left[kT\right]=K_{p}\theta \left[kT\right]+K_{d}\omega \left[kT\right]$, where $K_{p}$ and $K_{d}$ denote the proportional and derivative control gains, respectively, that are designed using the standard pole-placement technique. To do so, first a continuous-time model of the plant is obtained. The plant, composed of an armature-controlled DC motor, gearbox, and load (i.e., the motor arm, mount, and the smartphone), has a dynamic behavior that can be captured by a first-order transfer function from voltage input to the motor V(s) to the angular velocity of the motor Ω(s):

(1) $$\frac{\Omega (s)}{V(s)}=\frac{K}{\tau s+1}$$

where the values of the parameters are experimentally identified as K=1.5981 V and τ=0.029 s. Next, Equation (1) is represented in a state-space form and discretized to account for the fixed-rate sampling performed by the smartphone. Samplers and zero-order holds are introduced in the feedback and feedforward paths of the closed-loop (see Figure 3). Since the control data is transmitted to the host machine in the same TCP/IP packet as the sensor readings, which are used by the host machine only for data logging, the three samplers are assumed to share the same uniform sampling rate *T*. Next, for a desired closed-loop performance, the closed-loop bandwidth is determined that specifies an acceptable sampling rate, yielding the discretized state-space model below:

(2) x[(k+1)T]=Φ(T)x[kT]+Γ(T)u[kT]

y[kT]=Cx[kT]

where $x\left[kT\right]≜{\left[\theta [kT]\;\omega [kT]\right]}^{T}$ and the sampled-data state-transition and input matrices in Equation (2) (i.e., Φ(T) and Γ(T)) are obtained from the corresponding continuous-time state and input matrices (i.e., *A* and *B*) as delineated below and *C* is the system output matrix:

(3) $$\Phi \left(T\right)≜e^{AT},\;\Gamma \left(T\right)≜\int_{0}^{T}\Phi \left(T-\tau \right)Bd\tau$$

(4) $$A≜\left[\begin{array}{cc} 0 & 1\\ 0 & -\frac{1}{\tau } \end{array}\right],\;B≜\left[\begin{array}{c} 0\\ \frac{K}{\tau } \end{array}\right],\;C≜\left[\begin{array}{cc} 1 & 0\\ 0 & 1 \end{array}\right]$$

Next, these system matrices are used to design the PD controller gains. A more detailed derivation of the system modeling and control design for this smartphone-mounted testbed is provided in [35].

### 4.2. Inverted Pendulum Test-Bed

**Figure 4:**
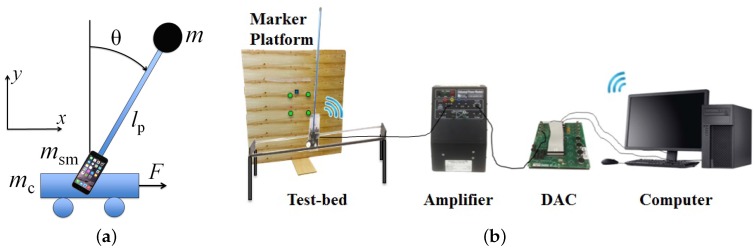
(**a**) A smartphone-mounted IPC system and (**b**) experimental setup with a PC-based DAC.

The second test-bed explored in this study is an educational IPC test-bed mounted with a smartphone (see Figure 4a). A cart is driven along a track by a DC motor, which is fitted with a potentiometer to measure the cart’s translation along the track. A 60.6 cm pendulum arm is pinned to the cart and uses an incremental optical encoder to measure its rotation. As in the first case, a power amplifier drives the DC motor using signals received from a PC through a DAC board, as shown in Figure 4b. An Apple iPhone 6 Plus, which has a 1.2 megapixel front-facing camera that supports frame rates up to 60 fps, is responsible for all sensing on the system. Although multimedia frameworks such as GStreamer that support the Real-Time Protocol (RTP) could be used to transmit the video, real-time video streaming necessitates a hardware encoding process that consumes large amounts of battery and introduces latency that could affect the stability of the closed loop. Thus, video is not transmitted in this research. Instead, efficient image processing algorithms are used to study the capacity of smartphones to process video frames themselves.

#### 4.2.1. Smartphone Measurement

**Figure 5:**
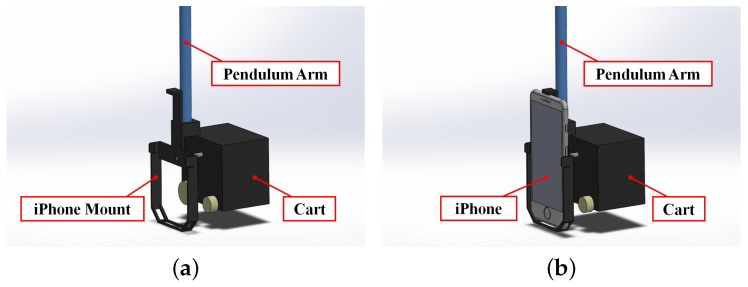
Design of a mount for the IPC system (**a**) without and (**b**) with a smartphone secured.

**Figure 6:**
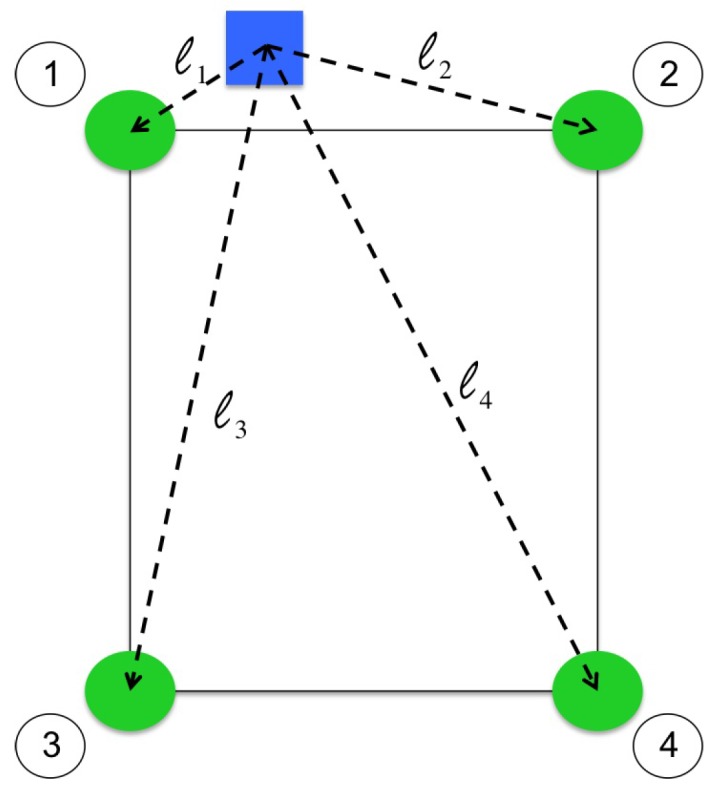
Pattern of markers on the platform in front of the IPC test-bed with $l_{1}<l_{2}<l_{3}<l_{4}$.

In this second case, both the rotational motion of the pendulum arm and the translational motion of the cart on the track must be sensed. Thus, the smartphone is rigidly mounted such that the centroidal axis normal to its screen is aligned with the rotational axis of the pendulum arm. In this way, the rotation of the smartphone about its axis corresponds to the pendulum arm rotation and the translation of the smartphone corresponds to the cart translation (see Figure 5). In front of the IPC test-bed, and in view of the front-facing camera of the smartphone from all possible states of the IPC, a platform is fitted with a known pattern of colored markers (see Figure 6). In real time, the smartphone processes video of the platform using a color segmentation approach to detect the image locations of the markers. Since the stability of the closed-loop system requires efficient sensing and processing implementations, the lowest supported image resolution is used (192 × 144) and markers are searched for in small neighborhoods of their previous detected locations. However, as will be discussed in Section 6, lowering image resolution will increase noise in the measurements.

Once the markers have been detected, the mobile application identifies each green marker by its relative position from the blue marker (see Figure 6). Then, using the four point correspondences between the 3D world coordinates of the markers (with respect to a coordinate system established with origin at the first green marker) and their 2D projected coordinates in the image, the relative pose between the plane containing the markers and the image plane of the smartphone camera is estimated [36]. This pose estimate is finally decomposed into rotational and translational components that correspond to the rotation and translation of the smartphone (and thus the IPC).

#### 4.2.2. System Modeling and Control

The vision-based feedback from the mounted smartphone provides measurements of only two of the four states needed for full-state feedback control of the IPC system. These measurements contain noise due to factors such as imperfections in image quality, scene illumination, and the color segmentation procedure. Noise associated with the detected centers of the markers will result in noisy cart position and pendulum angle measurements. Thus, a steady-state discrete-time Kalman filter is implemented to obtain estimates $\hat{x}$ of the state $x≜{\left[x\;\theta \;\dot{x}\;\dot{\theta }\right]}^{T}$, which includes two unmeasured states (i.e., the velocity of the cart and the angular velocity of the pendulum) as well as the two measured states (i.e., the cart position and the pendulum angle). This Kalman filter is implemented at each time step kT by propagating the following state estimation equation:

(5) $$\hat{x}\left[\left(k+1\right)T\right]=\Phi \left(T\right)\hat{x}\left[kT\right]+\Gamma \left(T\right)u\left[kT\right]+L\left(y\left[kT\right]-C\hat{x}\left[kT\right]\right)$$

where *L* is the Kalman gain and y[kT] is the measurement received from the smartphone. Moreover, analogous to the Equation (2), matrices Φ(T) and Γ(T) for the sampled-data system are obtained using matrices *A* and *B* for the continuous-time system as in Equation (3). For the IPC system, matrices *A*, *B*, and *C* are given as follows:

(6) $$A≜\left[\begin{array}{cccc} 0 & 0 & 1 & 0\\ 0 & 0 & 0 & 1\\ 0 & -\frac{mgl_{p}}{J_{\text{eq}}\gamma } & -\frac{1}{ml_{p}\gamma }\left(\frac{K_{m}^{2}K_{g}^{2}}{Rr^{2}}\right) & 0\\ 0 & \frac{(M+m)g}{J_{\text{eq}}\gamma } & \frac{1}{J_{\text{eq}}\gamma }\left(\frac{K_{m}^{2}K_{g}^{2}}{Rr^{2}}\right) & 0 \end{array}\right],\;B≜\left[\begin{array}{c} 0\\ 0\\ \frac{1}{ml_{p}\gamma }\left(\frac{K_{m}K_{g}}{Rr}\right)\\ -\frac{1}{J_{\text{eq}}\gamma }\left(\frac{K_{m}K_{g}}{Rr}\right) \end{array}\right],\;C≜\left[\begin{array}{cccc} 1 & 0 & 0 & 0\\ 0 & 1 & 0 & 0 \end{array}\right]$$

where $\gamma ≜\frac{m+M}{ml_{p}}-\frac{ml_{p}}{J_{\text{eq}}}$, $J_{\text{eq}}≜J_{\text{sm}}+ml_{p}^{2}$, $M≜m_{c}+m_{\text{sm}}$, and $J_{\text{sm}}≜m_{\text{sm}}\left(l_{\text{sm}}^{2}+w_{\text{sm}}^{2}\right)/12$. The physical quantities and numeric values of the model parameters are provided in Table 1. The state, input, an output matrices of Equation (6) result from the dynamic model of the SMLTB that is derived using the free-body diagram shown in Figure 4a with either Newton’s method or the Euler-Lagrange approach outlined in [32,37]. Moreover, sampled-data system’s state transition, input, and output matrices, (i.e., Φ(T), Γ(T), and *C*, respectively) are used to verify the observability and controllability of the system. Next, the matrix solutions to discrete-time algebraic Riccati equations are used to compute the Kalman filter gain and linear quadratic regulator gain for the SMLTB [38]. A more detailed derivation of the modeling, filtering, and control of this SMLTB is provided in [39].

### 4.3. Ball and Beam Test-Bed

**Figure 7:**
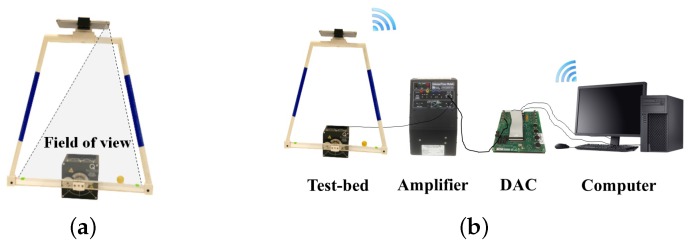
(**a**) Smartphone-mounted ball and beam and (**b**) experimental setup with a PC-based DAC.

The final test-bed explored in this study is a ball and beam system built using the motor test-bed described in Section 4.1, with a smartphone mounted to a beam using a 3D-printed plastic structure, as shown in Figure 7a. The motorized beam is driven using the same experimental setup as the previous two test-beds (see Figure 7b). An Apple iPhone 6 Plus uses its InvenSense MP67B 6-Axis MEMS IMU and 8-megapixel rear-facing camera to measure three out of the four states of the system.

#### 4.3.1. Smartphone Measurement

In this third case, both the rotational motion of the beam and the translational motion of the ball on the beam must be sensed by a smartphone. To detect ball position, the ball is painted yellow and a green marker is attached at each end of the beam. Each of the three colored objects is detected using the same color segmentation approach described in the previous case. Because the smartphone is mounted such that the user interface is accessible to the user (with its screen pointed up), the rear-facing camera is used to obtain vision-based measurements of the ball position on the beam. A 3D-printed trapezoidal mounting structure is constructed using PLA plastic to rigidly mount the smartphone at a height above the beam that allows the ball to be observed as it travels along the entire length of the beam, as shown in Figure 7a.

Knowledge of the smartphone’s rigid attachment to the structure is exploited to make the search for each of the colored objects more efficient, significantly reducing processing time and preserving stability of the closed-loop system. Since the locations of the green markers at the ends of the beam do not move relative to the camera, small square neighborhoods are used to search for these markers. Moreover, the search space for the ball is defined as a thin strip around the beam in the video frames. The normalized ball position along the beam is calculated from the distance, in pixels, between the image coordinates of the ball, $p_{b}=\left(x_{b},y_{b}\right)$, and the marker on the left end of the beam, $p_{\ell }=\left(x_{\ell },y_{\ell }\right)$, as well as the distance between the left end and right end of the beam, $p_{r}=\left(x_{r},y_{r}\right)$:

(7) $$x_{\text{norm}}=\frac{p_{b}-p_{\ell }}{p_{r}-p_{\ell }}$$

Equation (7) can be interpreted as the percentage of the beam’s length that the ball has traveled from left to right. Knowing the distance in real-world units between the markers, *l*, measurements for the ball position *x* are obtained with respect to the point halfway between the markers $x=(x_{\text{norm}}-0.5)\times l$.

The trapezoidal structure attached at the ends of the beam allows the beam, structure, and phone to be treated as a rigid body. Thus, the smartphone’s accelerometer and gyroscope can be used to capture the orientation of the beam. To measure angular orientation, readings of device attitude are generated after raw accelerometer and gyroscope data are processed by the device’s sensor fusion algorithms. Filtered data from the gyroscope are used as measurements of the beam’s angular velocity. Device attitude is expressed as the rotation between the device’s current reference frame and a reference frame formed using the direction of gravity obtained from the accelerometer. While the sampling rate of inertial measurements can be chosen to be as high as 100 Hz, the vision-based measurements are limited to the maximum supported frame rate of the iPhone 6 Plus’s rear-facing camera, which is only 30 Hz. To simplify the multi-rate control problem, the inertial and vision-based measurements are synchronized by choosing the sampling rate of the inertial measurements to be 90 Hz, an integer multiple of the vision-based measurements.

#### 4.3.2. System Modeling and Control

**Figure 8:**
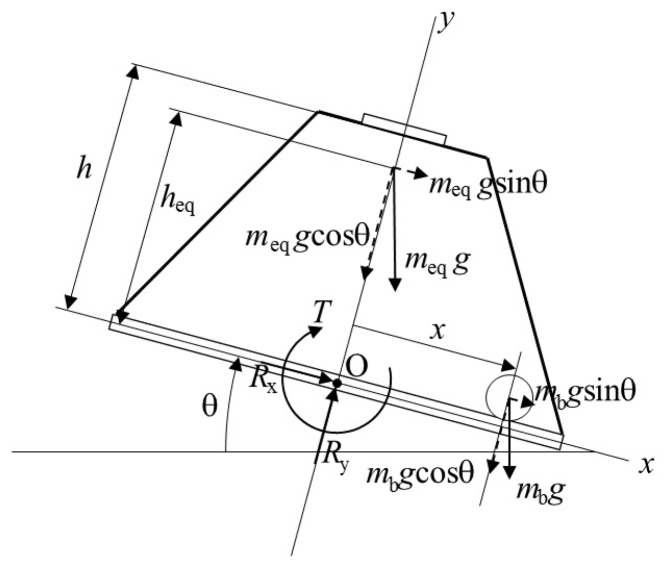
Free-body diagram of the smartphone-mounted ball and beam system.

To design a system that controls the ball and beam test-bed with visual and inertial feedback from a smartphone, the system is modeled in three separate parts: (1) the electromechanical behavior of the DC motor; (2) the rotational dynamics of the beam, mounted structure, and smartphone, which introduce nonlinear terms; and (3) the translational dynamics of the ball along a v-shaped groove on the surface of the beam. When modeling the motor, the electrical dynamics is neglected since its time constant is two orders of magnitude larger than that of the mechanical dynamics. The free-body diagram shown in Figure 8 aids in obtaining a model of the beam and ball dynamics. Note that with $m_{\text{sm}}$ denoting the mass of the smartphone with center of mass located at (0,h) and $m_{\text{st}}$ denoting the mass of the structure with center of mass located at $\left(0,\frac{2}{3}h\right)$, the equivalent mass of the smartphone and trapezoidal mounting structure can be denoted by $m_{\text{eq}}≜m_{\text{sm}}+m_{\text{st}}$ with its center of mass located at $h_{\text{eq}}≜\frac{hm_{\text{sm}}+0.67hm_{\text{st}}}{m_{\text{eq}}}$. After summing the moments about point O to solve for the beam’s angular acceleration, using the Euler-Lagrange approach to solve for the ball’s linear acceleration, and combining results with the motor’s electrical model, we obtain the following model of the system:

(8) $${\dot{x}}_{1}=x_{2},\;{\dot{x}}_{2}=\gamma \sin x_{3},\;{\dot{x}}_{3}=x_{4},\;{\dot{x}}_{4}=\mu \sin x_{3}-\sigma x_{4}+\delta u,\;y=x_{1}\;$$

where $x_{i}$, i=1,…,4, are components of $x≜{\left[x\;\dot{x}\;\theta \;\dot{\theta }\right]}^{T}$, $\gamma ≜\frac{m_{b}g}{I_{b}/r_{b}^{2}+m_{b}}$, $\mu ≜\frac{m_{\text{eq}}gh_{\text{eq}}}{I}$, $\sigma ≜\left(\frac{b_{t}}{I}+\frac{K_{b}K_{g}^{2}K_{T}}{IR_{a}}\right)$, and $\delta ≜\frac{K_{g}K_{T}}{IR_{a}}$. The numerical values for the physical parameters of the system are provided in Table 2. The nonlinear dynamics of Equation (8) are input-output linearizable. Thus, the change of variables $\xi ≜T\left(x\right)={\left[\begin{array}{cccc} x_{1} & x_{2} & \gamma \sin x_{3} & \gamma x_{4}\cos x_{3} \end{array}\right]}^{T}$ transforms Equation (8) into the normal form [40]:

(9) $$\dot{\xi }=A_{c}\xi +B_{c}\beta^{-1}\left(x\right)\left[u-\alpha \left(x\right)\right],\;y=C_{c}\xi$$

where:

$$A_{c}≜\left[\begin{array}{cccc} 0 & 1 & 0 & 0\\ 0 & 0 & 1 & 0\\ 0 & 0 & 0 & 1\\ 0 & 0 & 0 & 0 \end{array}\right],\;B_{c}≜\left[\begin{array}{c} 0\\ 0\\ 0\\ 1 \end{array}\right],\;C_{c}≜{\left[\begin{array}{c} 1\\ 0\\ 0\\ 0 \end{array}\right]}^{T}$$

$$\alpha \left(x\right)≜-\frac{1}{\delta \gamma \cos x_{3}}\left[-\gamma x_{4}^{2}\sin x_{3}+\gamma \cos x_{3}\left(\mu \sin x_{3}-\sigma x_{4}\right)\right],\;\beta \left(x\right)≜\frac{1}{\delta \gamma \cos x_{3}},\;x_{3}\neq \pm \frac{\pi }{2}$$

Since our control objective is for the ball position y(t)=x(t) to track a step command *r* issued by the user, an integrator state $\dot{\rho }=e$, where $e≜y-r=x_{1}-r$, is augmented to Equation (9) as in [40]:

(10) $$\dot{\xi_{a}}=A\xi_{a}+B\beta^{-1}\left(x\right)\left[u-\alpha \left(x\right)\right]$$

where:

$$A≜\left[\begin{array}{cc} A_{c} & 0\\ C_{c} & 0 \end{array}\right],\;B≜\left[\begin{array}{c} B_{c}\\ 0 \end{array}\right]$$

$$Y_{r}≜{\left[\begin{array}{cccc} r & 0 & 0 & 0 \end{array}\right]}^{T},\;\xi_{a}≜\left[\begin{array}{c} E\\ \rho \end{array}\right],\;E≜\xi -Y_{r}$$

The dynamics of Equation (10) are discretized using an analogous procedure as in Equation (3). Note that the mounted smartphone is used to provide measurements of three out of the four original states of the system Equation (9): the beam’s angular position and velocity, which are sampled at 90 Hz, and the ball’s position along the beam, which is sampled at 30 Hz. Thus, the objectives of the state estimation include: (1) estimating the unmeasured velocity of the ball; (2) filtering the noise corrupting the ball position measurement; and (3) integrating the measurements collected from disparate sensing sources at different rates. Thus, a partial-state estimator is designed that obtains estimates at 90 Hz for the ball position and velocity using measurements of ball position and dynamics of the ball driven by $\hat{u}=\sin x_{3}$. To handle the multirate estimation problem, the value of the ball position measurement at inter-sample periods must be chosen. Common approaches are setting the ball position measurement to either the value estimated by the dynamic model (equivalently, setting the observer gain L=0) or holding its value to the most recently measured value until a new measurement arrives. An experiment comparing the response using both approaches is conducted. A more detailed derivation of the modeling, estimation, and control for this SMLTB is provided in [41].

## 5. User Interfaces

**Figure 9:**
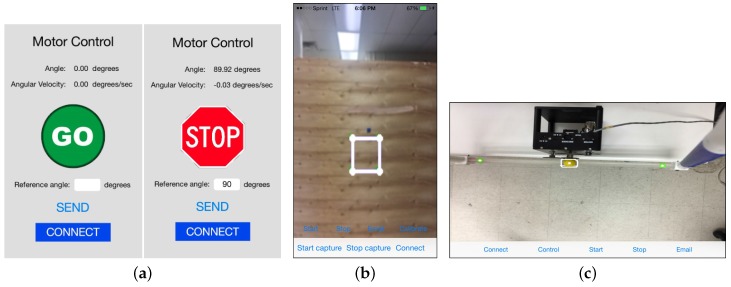
User interfaces for the (**a**) motor; (**b**) inverted pendulum; and (**c**) ball and beam test-beds.

The front end of mobile applications were developed to provide users with intuitive and engaging interfaces for interacting with the experiments outlined in the previous section. Supported interactions include the ability to wirelessly connect to the PC, start and stop control of the experiment, start and stop video capture, issue reference commands, calibrate sensor measurements, and collect valuable experimental data that users can email to themselves or to collaborators for further analysis. Useful visual feedback is provided to the user, such as the data collected by the application and the status of the image processing algorithm used to estimate the state of the systems. This allows users to easily calibrate and troubleshoot the systems. Furthermore, for the ball and beam system, users can tap on the image of the beam on the touch screen at the location where they would like the system to stabilize the ball. Screenshots of the mobile applications are shown in Figure 9.

## 6. Results

The prior sections have provided an overview of the development of three test-beds that utilize the on-board inertial sensors and cameras of mounted smartphones to measure motion. In addition to aforementioned challenges associated with integrating smartphones on laboratory test-beds, we must also examine the influence of noise, drift, and time delays on the smartphone-based measurements and their impact on the stability and performance of the system. These phenomena are associated with several factors, including the smartphone’s embedded hardware and software, the sensing modalities, the computational algorithms, and the wireless communication between the smartphone and the test-bed. Thus, several experiments are conducted to explore the effects of these factors on the behavior of the SMLTBs. We show that, in spite of the various challenges, the SMLTBs yield acceptable performances for use in engineering laboratory education.

### 6.1. DC Motor Test-Bed

#### 6.1.1. Inertial Measurement

One of the most critical factors in guaranteeing the closed-loop stability of the test-beds is ensuring that measurements are sampled at a rate that is sufficiently fast relative to the dynamics of the test-bed. A commonly used rule of thumb in digital control design is for the sampling rate to be faster than 30 times the closed-loop bandwidth frequency, $\omega_{b}$, which can be calculated from values of damping ratio *ζ* and natural frequency $\omega_{n}$ of the system dynamics [42]:

(11) $$\begin{array}{c} \omega_{b}=\omega_{n}{\left[\left(1-2\zeta^{2}\right)+\sqrt{4\zeta^{4}-4\zeta^{2}+2}\right]}^{1/2} \end{array}$$

By designing a controller that regulates the motor to a desired orientation within one second without any overshoot (ζ=0.9959 and $\omega_{n}=3.9282$ rad/s), the closed-loop bandwidth is calculated to be $\omega_{b}=2.5429$ rad/s (0.4047 Hz). Thus, the smartphone needs to provide attitude measurement at a rate faster than ≈12.2 Hz. Although mobile applications developed for the iPhones utilized in this study support sampling rates of up to 100 Hz for detecting high-frequency motions, such as impacts and quick shakes, processing of the data by the application may impose an upper bound on the sampling rate. After performing an experiment involving the collection of 350 samples, the mean motion data computation time on the smartphone is determined to be 244.4 μs with a standard deviation of 91.168 μs. This gives a 95% confidence interval of [239.13, 258.36] μs, which is fast enough to support a 100 Hz sampling rate, if desired. Although this low-bandwidth system supports a large range of sampling rates, note that the battery consumption by the sensors and communication module becomes substantially larger at faster sampling rates.

**Figure 10:**
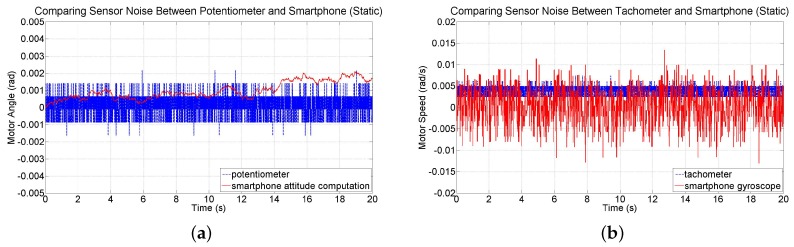
Comparisons between (**a**) the smartphone attitude sensing and potentiometer readings and (**b**) the smartphone gyroscope readings and tachometer readings while the motor is not being driven.

Drift errors associated with gyroscope data have been observed and documented [43]. Even commercially available gyros have been found to provide reliable estimates of orientation only for up to one minute of operation [44]. Fortunately, the test-beds investigated in this study are expected to be stabilized within just a few seconds. Nevertheless, the noise and drift characteristics of device attitude and angular velocity measurements are investigated by conducting two 20 s tests in which the smartphone’s IMU is sampled at 60 Hz and its readings are compared to readings from the potentiometer and tachometer sensors on the test-bed. The first test involved collecting sensor data while the motor is held at the zero orientation without being driven. The angular position data from the smartphone sensors and potentiometer are shown in Figure 10a for this static test. Smartphone attitude data has an average drift rate of 1.0297 × 10^−4^ rad/s (0.0059 deg/s). Angular velocity data from the gyroscope and the tachometer are shown in Figure 10b. Data from the tachometer has a slight bias; the mean of measurements from the gyroscope is −1.4439 × 10^−4^ rad/s (−0.0083 deg/s) compared to 0.0038 rad/s (0.2177 deg/s) from the tachometer. However, data from the smartphone is noisier and causes drift in attitude. The standard deviation of the measurements from the gyroscope is 0.0044 rad/s (0.2521 deg/s) and 7.0937 × 10^−4^ rad/s (0.0406 deg/s) from the tachometer.

**Figure 11:**
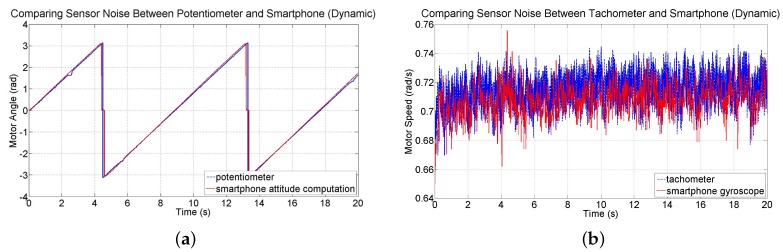
Comparisons between (**a**) smartphone attitude sensing and potentiometer readings and (**b**) smartphone gyroscope readings and tachometer readings while the motor is driven at constant speed.

A second test involved collecting data while the motor is driven at a constant speed. The angular position measurements from the smartphone and from the potentiometer of the test-bed are shown in Figure 11a. At the beginning of the run, the difference between the angular position readings is 0.0030 rad (0.1719 deg) and at the end of the run, the difference is −0.0174 rad (−0.9969 deg). Over the course of the run, the mean difference between the two sensors is −0.0247 rad (−1.4152 deg). These results indicate a sufficiently small error as the motor is driven for a relatively long time period. Measurements from the gyroscope and from the tachometer are shown in Figure 11b. The mean of the angular speed measurements from the gyroscope is 0.7070 rad/s (40.5081 deg/s) compared to 0.7163 rad/s (41.0410 deg/s) from the tachometer, indicating a difference of just 0.0093 rad/s (0.5329 deg/s). The standard deviation of the measurements from the gyroscope is 0.0113 rad/s (0.6474 deg/s) and from the tachometer 0.0107 rad/s (0.6131 deg/s), showing that the two sensors have noise of comparable magnitude.

#### 6.1.2. Communication

To test the communication between a smartphone and a test-bed over a wireless network, a command is issued on the PC to ping the smartphone and another PC. After sending 100 echo requests with 32 byte packets, the average round trip times for the smartphone and the other PC are determined to be 78.11 ms and 30.79 ms, respectively. A paired *t*-test is conducted to test whether the two sets come from distributions with equal means. With a 95% confidence level, the null hypothesis can be rejected (with t(99)=2.324,p=0.022<0.05,95%CI=[6.927,87.713]). Thus, communication with a smartphone experiences larger latency than a typical PC on the same network. Network latency results in time delays between data collected by the sensors on the test-bed and the embedded sensors of the smartphone. These time delays in the closed-loop system can vary significantly, which can cause degradation in system stability and performance and complicate analysis and control design. Several control methodologies have been used over the last several decades to compensate for such effects, spanning the use of Smith predictors, optimal stochastic methods, fuzzy logic, and queuing and buffering. However, with a mean one-way communication delay of 39.055 ms, average delays in the system are only between 0 and 4 sampling periods, depending on the sampling rate used. As will be shown, this amount of delay is negligible when controlling the motor-based test-beds, whose bandwidths are sufficiently low.

#### 6.1.3. System Response

**Figure 12:**
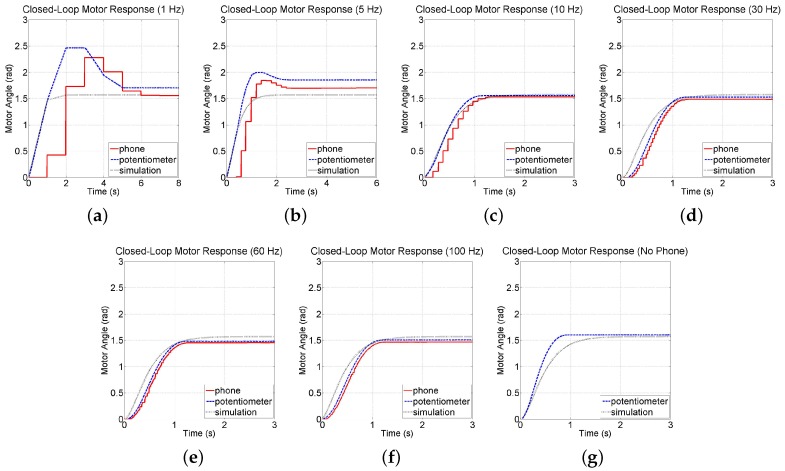
Closed-loop responses of the motor test-bed using the sensors from the (**a**–**f**) smartphone at various sampling rates and (**g**) test-bed.

To explore the feasibility of controlling the motor test-bed using attitude and speed measurements collected by a smartphone and of running the PD-control algorithm directly on the smartphone, trials are conducted in which the motor is given a step command of 90 deg (1.5708 rad). Figure 12a–f show the motor’s closed-loop response at several sampling rates. By plotting the angular position reported by the smartphone alongside those by the potentiometer, one can visualize the communication delay between the smartphone and computer as well as the drift in the smartphone sensor data. Note that simulation results are also plotted alongside the experimental results. For comparison, Figure 12g shows the response when the PD controller is implemented on the PC using feedback from the potentiometer and tachometer sensors of the test-bed. Table 3 shows the percent overshoot and settling time for each of the experimental responses. These results indicate an improvement in the responsiveness of the system as the sensors are sampled faster. Note that a loss in performance is observed from the motor when the smartphone-based controller is run slower than 10 Hz. This is because sampling at 5 Hz and 1 Hz, on the order of the closed-loop bandwidth frequency, leaves a large amount of inter-sample behavior uncaptured by the smartphone. Finally, each response exhibits varying degrees of steady-state error, which are attributed to friction in the motor and gearbox causing a deadzone in the motor’s sensitivity around 0 V that can be overcome by introducing an integral term to the controller. Note that the response of the motor at each sampling rate is consistent and varies little between sampling rates.

To confirm that time delays introduced by wireless communication may be neglected in the design of the SMLTB, a simulation of the sampled-data model is run with a constant delay of 39.055 ms introduced (the mean one-way communication time measured in Section 6.1.2). As seen in Figure 12, the experimental results match the simulated responses quite well, indicating that delays of 0–4 sampling periods are not significant when controlling a system with such a low bandwidth. Results of experiments with the IPC test-bed will explore the response of a high-bandwidth plant that utilizes smartphone sensing.

### 6.2. Inverted Pendulum Test-Bed

Unlike the motor test-bed, the IPC has nonlinear, high-bandwidth, open-loop unstable, and underactuated dynamics. In the vision-based control of such systems, limitations in image resolution, image processing speed, and frame rate supported by the camera of the smartphone can introduce noise and time delays that can degrade system stability and performance. A series of experiments are performed to obtain insights into the relationships between these factors.

#### 6.2.1. Vision-Based Measurement

As seen with the motor test-bed, sampling rate has an important impact on stability and performance. In the vision-based control of a SMLTB, this sampling rate is equivalent to the frame rate of the smartphone camera. Using Equation (11), the closed-loop bandwidth of the IPC test-bed is calculated to be 7.0995 Hz (44.6072 rad/s), suggesting a frame rate of ≈213 Hz, or higher. This rate is not achievable with our SMLTB, thus the highest achievable frame rate should be used for this high-bandwidth system. Although the standard frame rate of most smartphones is 30 fps, a higher frame rate can be achieved at the cost of lower image resolution. A resolution of 192 × 144 is chosen for experiments with the IPC test-bed since it is the lowest available and it allows the fastest frame rate, 60 fps, supported by the iPhone 6 Plus. The time required to process each frame introduces a delay between the instant that the visual data is captured by the camera and the instant that the measurement interpreted from that data becomes available. With a 60 fps rate, a processing time of up to 16.667 ms is acceptable, however large processing times can cause noticeable effects on stability or performance. Moreover, if the processing time exceeds the upper bound per frame, subsequent frames are discarded, causing information about the state of the system to be delayed until processing is complete. To characterize the effect of image resolution on computation time, frames are captured at 10 fps to avoid discarding any frames. Table 4 shows the computation times obtained for a variety of image resolutions. After performing an experiment in which 200 measurements are collected at a resolution of 192 × 144 (the lowest resolution supported by the iPhone 6 Plus), the mean computation time is found to be 5.09 ms with a standard deviation of 0.3609, which is fast enough to support a 60 fps rate. Thus, this resolution is chosen for the stabilization of the IPC (T=1/60 s).

**Figure 13:**
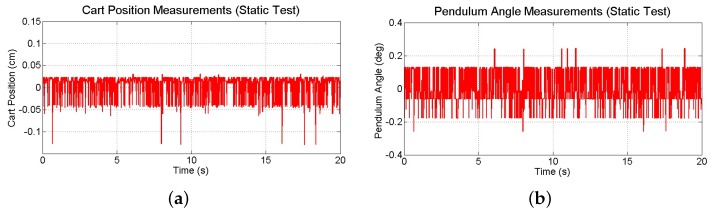
Measurements of (**a**) cart position and (**b**) pendulum angle collected during a static test.

Although lower image resolutions allow for faster processing times, this comes at the expense of increased measurement noise, which may degrade the performance of the system. To investigate the effect of image resolution on the noise characteristics of the vision-based measurements, raw data is collected while the IPC is kept in its stable equilibrium configuration (see Figure 13). Over the course of 20 s, standard deviations of cart position and pendulum angle measurements are determined to be 0.0258 cm and 0.1102 deg, respectively. Although this noise is larger than that observed with higher image resolutions, it will be shown to be acceptable for intended purpose of the smartphone.

**Figure 14:**
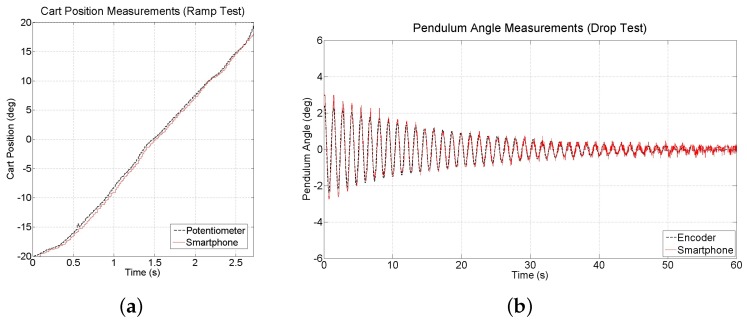
Measurements of (**a**) cart position collected during a ramp test and (**b**) pendulum angle collected during a drop test.

To further evaluate the accuracy and noise associated with the vision-based measurements, two additional experiments are conducted to compare these measurements to those obtained from the potentiometer connected to the motor and the encoder connected to the pendulum. The first experiment, a ramp test, examines cart position measurements as a ramp reference is applied to the cart position for approximately 3 s. Results (see Figure 14a) confirm that the smartphone can accurately measure the position of the cart, although the measurements suffer from a time delay of approximately 30 ms. The second experiment, a drop test, is run by lifting the pendulum arm a small amount from its stable equilibrium and dropping it to allow it to swing to a stop. Results (see Figure 14b) show that the smartphone measurements have acceptable levels of accuracy, however suffer from the same time delay as the position measurements.

#### 6.2.2. System Response

**Figure 15:**
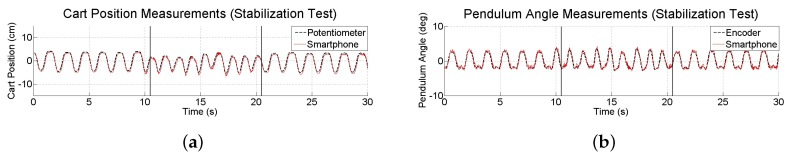
Experimental response of (**a**) cart position and (**b**) pendulum angle before, during, and after the use of vision-based measurements from the mounted smartphone.

To investigate the response of the IPC test-bed as it is controlled using vision-based measurements from the camera of the mounted smartphone, the IPC is first controlled using measurements from the standard potentiometer and encoder sensors on board the test-bed, as shown in Figure 15. After approximately 10.5 s, the source of the measurements is switched to the vision-based measurements of the smartphone. Then, after 10 s, the measurement source is switched back to the potentiometer and encoder. The response of the IPC before, during, and after the use of the vision-based measurements is shown in Figure 15. The system response remains stable with the smartphone measurements, and resembles the response with the standard sensors. Small variation between the response using smartphone camera and that using the test-bed sensors can be attributed to the small amounts of delay and noise introduced by the smartphone sensing. With more robust computer vision techniques and smartphones that can support faster frame rates and higher image resolutions, the performance of high-bandwidth systems like the IPC with mounted smartphones is expected to improve significantly.

### 6.3. Ball and Beam Test-Bed

The addition of the smartphone can add new challenges to the modeling, measurement, filtering, and control problems related to SMLTB. In the case of the ball and beam test-bed, nonlinear dynamics are introduced by the weight added on the beam, and limitations in image resolution, computation speed, and frame rate are again encountered, this time with the back-facing camera of the smartphone. A set of experiments show that, in spite of the challenges, the SMLTB yields acceptable stability and performance in tracking step references as compared to simulations and a benchmark experimental response obtained using a conventional measurement approach.

#### 6.3.1. Measurement Precision

**Figure 16:**
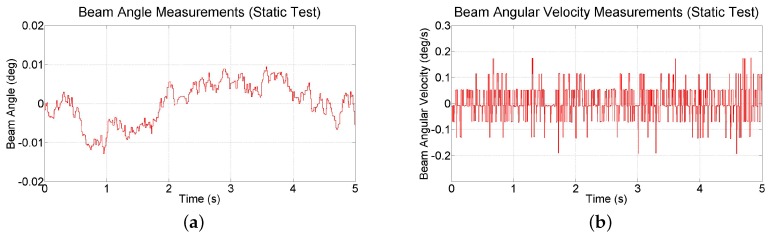
Measurements of (**a**) beam angle and (**b**) angular velocity collected during a static test.

To explore the noise characteristics of the inertial measurements provided by the smartphone, raw data is collected at 90 Hz for 5 s while the test-bed is at rest at its zero state (see Figure 16). A small amount of drift is observed in the orientation measurements. The range of beam angle measurements is 0.022 deg and variance of angular velocity is 0.0041 (deg/s)^2^. For the image-based vision approach on the smartphone, frame rate of back-facing camera is restricted to 30 fps (33 ms processing time). Applying Equation (11), the bandwidth of the ball and beam test-bed is calculated to be 0.8848 Hz (5.559 rad/s), suggesting a frame rate of ≈26.5 Hz, or higher. Thus, although the back-facing camera of the iPhone 6 Plus only supports frame rates of 30 fps, this rate is sufficient for the ball and beam test-bed. At this frame rate, the highest allowable image resolution is 640 × 480 before the computation time exceeds the allowable range. An experiment indicates that at this resolution, the mean computation time is 30.24 ms, with a standard deviation of 0.6541 ms, and that the ball position can be measured to within 0.705 mm. This measurement resolution is approximately 25 times more coarse than the resolution of 0.028 mm that has been obtained with a measurement system consisting of a linear membrane potentiometer, an operational amplifier circuit, and a 14-bit analog-to-digital converter [45]. While a lower image resolution reduces processing time, it also reduces precision by increasing the level of noise in the measurements. Alternatively, a higher image resolution improves precision, but also increases processing time, causing latency that can destabilize the system. For example, experiments in which the image resolution was lowered to 352 × 288 degraded measurement precision and experiments in which the image resolution was increased to 1280 × 720 yielded large computation delays, both of which rendered the system unstable.

#### 6.3.2. System Response

**Figure 17:**
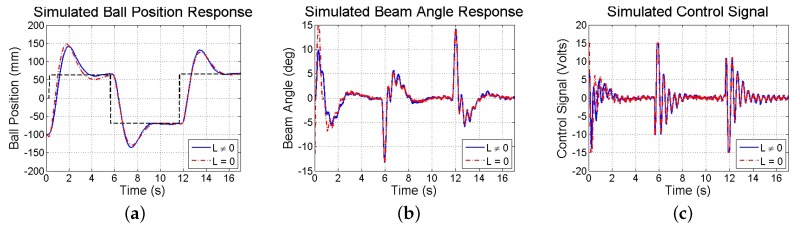
Simulated response of (**a**) ball position; (**b**) beam angle; and (**c**) control computed to step commands using two methods to estimate ${\hat{x}}_{p}$.

**Figure 18:**
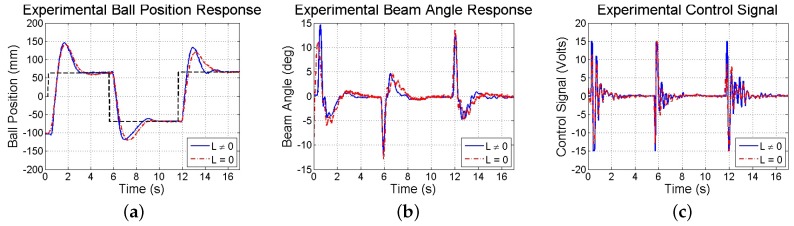
Experimental response of (**a**) ball position; (**b**) beam angle; and (**c**) control computed to step commands using two methods to estimate ${\hat{x}}_{p}$.

**Figure 19:**
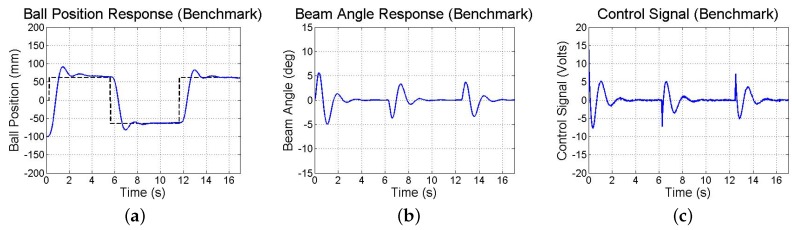
Experimental response of (**a**) ball position; (**b**) beam angle; and (**c**) control action obtained using conventional sensors.

Limitations in resolution, precision, and sampling rate introduced by the smartphone’s visual and inertial sensors have consequences on the test-bed’s closed-loop response. Thus, simulations and experiments are performed to investigate the stability and performance of the smartphone-mounted ball and beam test-bed in response to reference commands. First, a simulation is run taking into account experimentally observed sensor noise, computation and communication delays, measurement resolution, sampling rates, and actuator saturation. Both methods of state estimation (discussed in Section 4.3.2) are used and found to be comparable. The resulting closed-loop responses are shown in Figure 17. Next, in experiments, the same step commands generated from user taps on the touchscreen to drive a simulation are used to drive the actual test-bed utilizing the smartphone, whose responses are shown in Figure 18. Furthermore, Figure 19 illustrates the response of the benchmark system that utilizes a conventional sensing approach (an encoder and tachometer to measure the beam angle and angular velocity, respectively, and a linear membrane potentiometer to measure the ball position, as described in [45], all sampled at 1000 Hz). Although the system maintains stability and settles in approximately 3 s, the ball exhibits large overshoots as compared to the benchmark response due to limitations in smartphone-based sensing as discussed above.

### 6.4. Battery Consumption

To realize SMLTBs, researchers and learners mount to test-beds their personal devices and interact with them for extended periods. This necessitates a consideration of battery consumption of smartphones during operation. To investigate battery consumption rates, each SMLTB is run 3 times for 10 min using an iPhone 5 and an iPhone 6 Plus starting with a 100% charged battery. During each trial, all background applications and processes are closed so as not to affect results. Table 5 shows the average battery consumption rate measured over three trials for each combination of smartphone and test-bed, in units of minutes taken for the battery power to drop by 1% (smaller numbers indicate faster battery drain). Measurements are collected from the DC motor with inertial sensors being sampled at 100 Hz sampling rate. Results show that the IPC, which utilizes the front-facing camera of the smartphone at 60 Hz, drains battery much faster than the DC motor, indicating that the camera drains battery faster than the IMU. Next, the ball and beam test-bed, which utilizes both the back-facing camera of the smartphone at a 30 Hz sampling rate and the IMU at a 90 Hz sampling rate, drains battery slightly faster than the IPC. Note that the iPhone 6 Plus, which runs on a 1810 mAh battery compared to the 1440 mAh battery of the iPhone 5 and contains a more power efficient processor, drains battery slower than the iPhone 5. The results of Table 5 are promising since the average activity with the SMLTBs lasts approximately 30 min, even a 3-year old iPhone 5 whose battery has been recharged many times will lose only 14% of its battery during the course of an average activity with the most power draining experiment.

## 7. Conclusions

Smartphones are steadily becoming the primary personal computer in people’s lives. Since they already contain the sensing, storage, computation, and communication capabilities necessary to develop solutions, smartphones are uniquely suited as platforms for laboratory research and education. To inform developers and educators of the feasibility and performance of such platforms, this paper investigated the mounting of smartphones to laboratory test-beds to provide portable, economic, and engaging measurement and feedback control solutions. To explore the extent to which mounted smartphones may be employed to measure and control motion, and to address challenges associated with integrating smartphone-based sensing and computation into the system, three smartphone-mounted test-beds were developed and validated. Specifically, a smartphone’s inertial sensors, front-facing camera, and its inertial sensors and back-facing camera, were used with a DC motor, IPC, and ball and beam test-beds, respectively, for measurement and control. SSCC capabilities of the smartphone were put to the test to drive the system. Results from experiments validate the feasibility of smartphones as measurement and control platforms. With the release of mobile devices with even more powerful processing, higher resolution sensing, and faster communication, we expect to see their further incorporation in embedded motion sensing and control applications. To successfully achieve robust applications with disparate models of smartphones, future work will consider the design of mobile applications whose measurement and control algorithms adapt to differences in hardware (e.g., sensor specifications, size and weight of the device, etc.), as well as interfaces that adapt to different users. Furthermore, future efforts will explore techniques to handle more complex measurement and control tasks, whose stability and performance requirements test the limits of the hardware and software of SMLTB. Finally, future research will consider the educational effectiveness and user experiences associated with the use of mounted smartphones in science and engineering laboratories. Videos of the SMLTBs in action can be found at [46]. 

## Figures and Tables

**Table 1:** Numerical values for physical parameters of the IPC system.

Physical Quantity	Symbol	Numerical Value	Units
Cart mass	$m_{c}$	0.815	kg
Pendulum mass	*m*	0.210	kg
Smartphone mass	$m_{\text{sm}}$	0.172	kg
Pendulum length	$2l_{p}$	2 × 0.303	m
Cart wheel radius	*r*	0.0254/4	m
Smartphone length	$l_{\text{sm}}$	0.1581	m
Smartphone width	$w_{\text{sm}}$	0.0778	m
Gravitational constant	*g*	9.8	m/s^2^
DC motor resistance	*R*	2.6	Ω
Motor constant	$K_{m}$	0.00767	
Gear ratio	$K_{g}$	3.7	

**Table 2:** Numerical values for physical parameters of the ball and beam test-bed.

Physical Quantity	Symbol	Numerical Value	Units
Ball mass	$m_{b}$	0.007	kg
Smartphone mass	$m_{\text{sm}}$	0.172	kg
Structure mass	$m_{\text{st}}$	0.217	kg
Ball radius	$r_{b}$	0.013	m
Smartphone height	*h*	0.5	m
Gravitational constant	*g*	9.8	m/s^2^
Moment of Inertia	*I*	0.06711	kg·m^2^
Back-emf constant	$K_{b}$	0.00768	Volt·s/rad
Motor torque constant	$K_{T}$	0.00768	N·m/Amp
Friction coefficient	$b_{t}$	0.05	N·m·s
DC motor resistance	$R_{a}$	2.6	Ω
Gear ratio	$K_{g}$	14	

**Table 3:** Motor response performance using the digitally redesigned PD-control implementation.

Sampling Rate	Maximum Overshoot (%)	Settling Time (s)
no phone	0.00	0.798
100 Hz	0.00	1.096
60 Hz	0.00	1.128
30 Hz	0.00	1.140
10 Hz	0.00	1.216
5 Hz	8.12	2.188
1 Hz	46.23	5.986

**Table 4:** Effect of image resolution on computation time and measurement noise.

Image Resolution	Mean Computation Time (ms)	Computation Time SD (ms)	Cart Position SD (cm)	Pendulum Angle SD (deg)
192×144	5.09	0.3609	0.0258	0.1102
640×480	16.4301	0.5546	0.0112	0.0391
1280×720	39.7369	1.3213	0.0056	0.0194

**Table 5:** Average battery consumption rate (min/%) of each mounted smartphone and test-bed.

Test-Bed	iPhone 5 Consumption Rate	iPhone 6 Plus Consumption Rate
DC motor	4.18	6.12
IPC	2.58	4.62
Ball and beam	2.16	4.19

## Supplementary Materials

The following are available online at http://www.mdpi.com/1424-8220/16/8/1331/s1, Video S1: Wireless networked Control of a Motor testbed; Video S2: Stabilizing ball and beam test-bed; Video S3: Visual servoing of an inverted pendulum.

## Abbreviations

The following abbreviations are used in this manuscript:

SSCC	Sensing, storage, computation, and communication
SMLTB	Smartphone-mounted laboratory test-bed
IPC	Inverted pendulum on cart
IMU	Inertial measurement unit
PC	Personal computer
DAC	Data acquisition and control
PD	Proportional and derivative
fps	frames per second
